# Supplementary material for: The interactome of intact mitochondria by cross-linking mass spectrometry provides evidence for coexisting respiratory supercomplexes
Source: Mol Cell Proteomics. 2017 Dec 8;17(2):216–32. doi: 10.1074/mcp.RA117.000470 (PMC5795388; doi:10.1074/mcp.RA117.000470)
Supplement: Supplemental Data [file supp_RA117.000470_133922_0_supp_23978_fzffwf.zip › spectra_annotation/mito_DR_spectra_annotation/134-1-8-1-7-1.pdf]

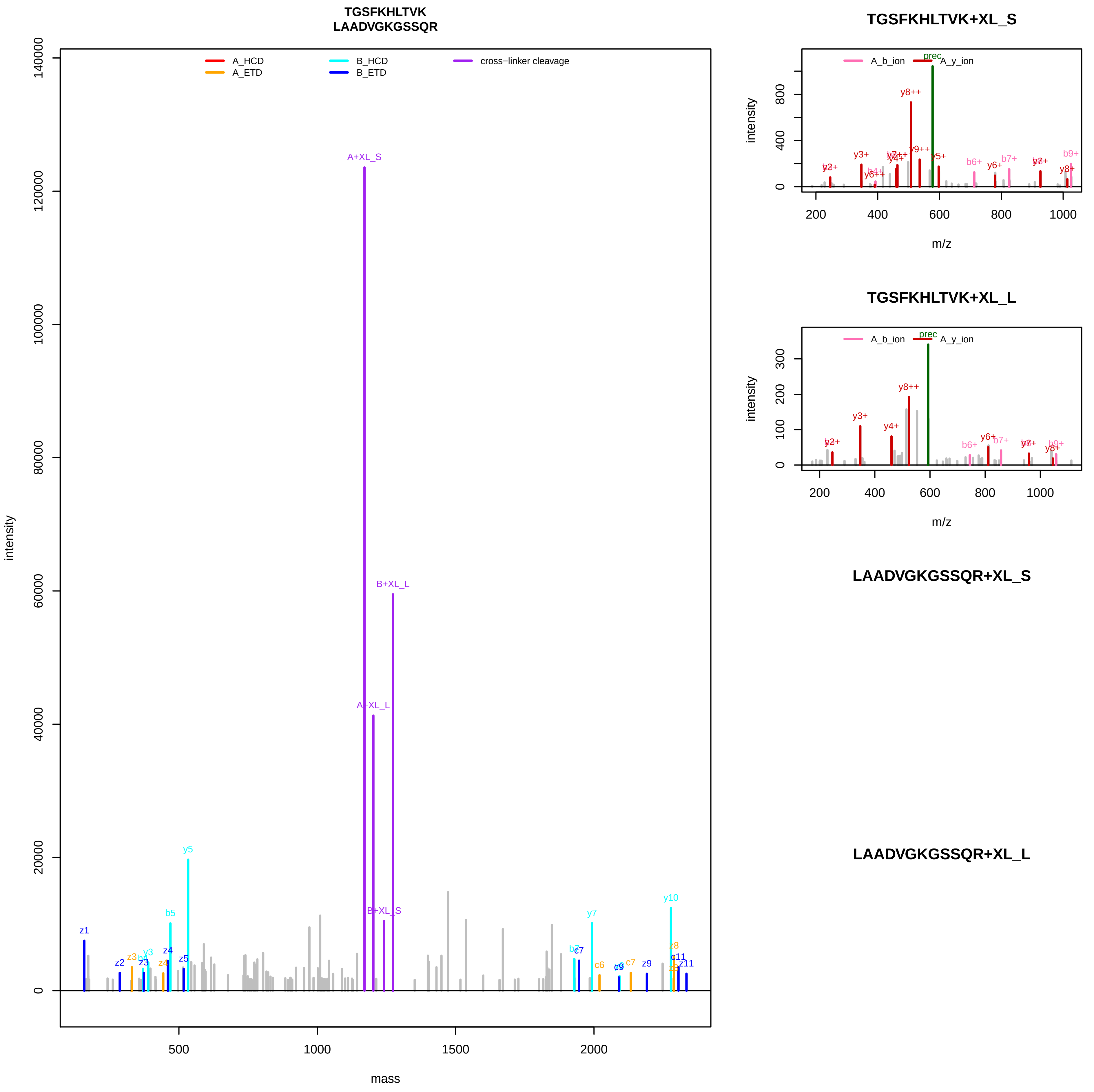
TGSFKHLTVK
LAADVGKGSSQR
A_HCD
A_ETD
B_HCD
B_ETD
cross−linker cleavage
A+XL_S
B+XL_L
A+XL_L
B+XL_S
intensity
mass
z1
z2
z3
z4
z5
y3
y5
b5
y7
y10
c7
c6
c9
z9
z8
c11
z11
TGSFKHLTVK+XL_S
TGSFKHLTVK+XL_L
A_b_ion
A_y_ion
prec
y2+
y3+
y4+
y5+
y6+
y7+
y8+
y8++
y9++
y6++
b6+
b7+
b9+
m/z
LAADVGKGSSQR+XL_S
LAADVGKGSSQR+XL_L
